# Co‐infection of dengue, scrub typhus, and typhoid during dengue outbreak in Nepal, 2022: A case report

**DOI:** 10.1002/ccr3.7080

**Published:** 2023-03-14

**Authors:** Bibek Raj Bhattarai, Rajshree Bhujel, Sushant Pokhrel, Abhishek Mishra, Anamika Priyadarshinee

**Affiliations:** ^1^ Department of Molecular Biology Modern Diagnostic and Research Center Kathmandu Nepal; ^2^ Department of Clinical Pathology Nepal Lab House Kathmandu Nepal; ^3^ Central Department of Microbiology Tribhuvan University Kathmandu Nepal; ^4^ Manmohan Memorial Institute of Health Sciences Kathmandu Nepal; ^5^ Tribhuvan University Teaching Hospital Kathmandu Nepal; ^6^ Department of Pathology KIST Medical College and Teaching Hospital Lalitpur Nepal

**Keywords:** co‐infection, dengue, dengue outbreak, scrub typhus, typhoid fever

## Abstract

In midst of the recent dengue outbreak in Nepal, in 2022, the risk of co‐infection increases and may lead to fatal outcomes if the diagnosis of multiple infections is delayed. Thus, all available diagnostic approaches must be taken to decrease the burden of illness and lessen mortality.

## 1 INTRODUCTION

Dengue is a tropical disease caused by the dengue virus transmitted by *Aedes aegypti* and *Aedes albopictus* mosquitoes. It is particularly prevalent in tropical and subtropical regions and has been expanding into new geographic areas throughout time.^1^ Scrub typhus, which is caused by the bacteria *Orientia tsutsugamushi*, is another disease to be concerned about due to its endemicity in Nepal. The lack of distinct clinical symptoms and diagnostic resources, scrub typhus is frequently under‐diagnosed.^2^ Another endemic febrile sickness in Nepal is typhoid fever; the causative agent *Salmonella typhi* causing multi‐systemic illness when contaminated food and water are consumed.^3^

Monsoon and post‐monsoon are the high‐risk seasons for spreading typhoid, dengue, and scrub typhus.^4^ Even though the co‐infections among the organisms are rare due to multiple etiological agents and/or vectors involved, the probability cannot be neglected. Here, we present a case of 58‐year‐old female from Dang, a district with a subtropical climate, having co‐infection with dengue, typhoid fever, and scrub typhus during a dengue outbreak in Nepal.

## 2 CASE REPORT

### 2.1 Clinical presentation

A 58‐year‐old woman presented with a continuous fever that persisted for 16 days. The patient was last well 16 days before presentation when she developed a fever of 103°F that was not accompanied by chills or rigor. She also reported experiencing 2 weeks of progressively worsening epigastric discomfort accompanied by a feeling of abdominal fullness. There was no history of rashes, chest pain, coughing, loose stools, nausea/vomiting. Additionally, the patient also stated that she had no prior history of thyroid disease, pulmonary TB, diabetes mellitus, or hypertension. She said that she does not smoke, although she does consume 200 mL of alcohol twice daily. The most recent intake was about 2 weeks ago.

### 2.2 History

The patient resides in the Dang district of Nepal, an inner belt of the Terai region, which has a subtropical climate. Her primary occupation was of a farmer and worked in agricultural fields where mites are more common. She was also active in household work. Additionally, she experienced the onset of her symptoms during September 2022 which marked post‐monsoon season in Nepal.

On examination, the patient was presented as conscious, pale, icterus, and had a low‐grade fever of 100.5°C along with bilateral pitting edema, oral ulcer, and abdominal tenderness in the epigastric region. Vital checkup of the patient presented hypotension (BP = 80/60 mmHg), tachycardia (PR = 116 beats per minute), Respiratory rate of 20 beats per minute, low‐level SPO_2_, that is, 89% in the room atmosphere.

### 2.3 Diagnostic tests and treatment

Considering the recent outbreak of dengue in Nepal in 2022 and based on the patient's presenting symptoms, tests for dengue (NS1 and IgG/IgM), and *S. typhi* IgG/IgM were requested along with routine hematological and biochemical parameters, urine culture and routine examination, and blood culture. Results revealed dengue IgM positivity along with thrombocytopenia (107,000/μL), which are consistent with dengue infection. Moreover, bilirubin (total/direct: 2.6/1.6) and liver enzymes (serum glutamate pyruvate transaminase: 133 U/L, serum glutamate oxaloacetate transaminase: 250 U/L, alkaline phosphatase: 526 U/L) were elevated along with subsequent hypoalbuminemia (2.4 g/dL). The *S. typhi* IgM test were positive, indicating possible concurrent *S. typhi* and dengue infection. Urine and blood culture tests were negative despite routine examination reporting an increase in pus and epithelial cells. The patient was immediately given hydration therapy with crystalloid fluid and 100 mg hydrocortisone. The patient received three doses of a 1000 mg paracetamol tablet each day and injections of 1000 mg ceftriaxone twice daily. Domperidone 10 mg and ondansetron 4 mg were given to the patient to treat the nausea and vomiting that accompanied indigestion. Tables 1 and 2 document the serological testing and subsequent hematological as well as biochemical profile, respectively, following patient hospitalization.

**TABLE 1:** Serological tests of the patient demonstrating infection with multiple micro‐organisms.

During admission		During follow‐up	
Rapid dengue	NS_1_ Ag‐negative IgM Ab‐positive IgG Ab‐negative	Rapid dengue	NS_1_ Ag‐negative IgM Ab‐negative IgG Ab‐positive
		Rapid *Salmonella typhi*	IgM Ab‐negative IgG Ab‐positive
		Rapid scrub typhus	IgM Ab‐positive IgG Ab‐negative
Rapid *S. typhi*	IgM Ab‐positive IgG Ab‐negative	Rapid *Leptospira*	IgM Ab‐negative IgG Ab‐positive
		Brucella total Ab test	Positive
		Rapid leishmania (K‐39) Ab	Negative
		Smear for malarial parasite	Not seen

**TABLE 2:** Hematological, biochemical, and urine analysis of patient during admission and subsequent follow‐up until discharge.

Laboratory parameters	Admission (Day 1)	Follow‐up I (Day 4)	Follow‐up II (Day 6)	Follow‐up III (Day 7)	Follow‐up IV (Day 8)	Discharge (Day 10)
Hematological						
Hemoglobin (g/dL)	12.3	11.8	10.0	10.6	10.1	12.4
Hematocrit (%)	29.9	33.7	27.3	29.0	29.0	35
RBC (million/cumm)	4.2	4.4	3.63	3.7	3.8	4.41
MCV	71.2	76.6	75.21	78.3	78.00	79.3
MCH	31	27.05	27.55	28.65	27.20	27.2
MCHC	43.6	35.31	36.63	36.55	35.00	34.3
Total leucocyte count (cells/cumm)	8100	9100	6400	5400	5300	4400
Differential leucocyte count (N/L/M/E/B)	81/11/03/05/00	79/16/04/01/00	39/54/05/02/00	24/68/06/02/00	26/65/06/03/00	28/61/05/06/00
Platelets	107,000	231,000	123,000	128,000	308,000	492,000
Biochemistry						
Bilirubin (mg/dL) Total/direct	2.6/1.6	4.04/2.70	–	2.5/1.7	–	0.74/0.32
SGPT (U/L)	133	130	–	200	–	19
SGOT (U/L)	250	289	–	248	–	16
ALP (U/L)	526	845	–	1602	–	325
Total protein (g/dL)	6.0	6.15	–	5.9	–	7.15
Albumin (g/dL)	2.4	2.27	–	2.10	–	3.53
Urine analysis						
Color	Dark yellow	Yellow	–	–	–	–
Transparency	Turbid	Slightly turbid	–	–	–	–
pH	Acidic	Acidic	–	–	–	–
Sugar	Negative	Negative	–	–	–	–
Protein	Trace	Trace	–	–	–	–
Pus cells	6–8/hpf	1–2/hpf	–	–	–	–
Epithelial cells	4–6/hpf	2–3/hpf	–	–	–	–
Red blood cells	0–1/hpf	Not seen	–	–	–	–
Casts	Moderate granular cast seen	Few granular cast seen	–	–	–	–
Crystal	Not seen	Not seen	–	–	–	–

After being hospitalized and treated with ceftriaxone and paracetamol, blood tests conducted on Day 4 showed normal platelet count and total white blood cell. However, on Day 6, decrease in platelets was observed along with abnormal blood parameters, leading to the follow‐up tests for dengue IgG/IgM and *S. typhi* IgG/IgM as well as several tests for tropical fever panel (scrub typhus IgG/IgM, *Leptospira* IgG/IgM, tests for total *Brucella* Ab, K‐39 total Ab test for Leishmaniosis, and smear for the malarial parasite). Acute infection of scrub typhus with IgM positivity was reported despite having no physical signs of eschar as seen in patients with scrub typhus infection. Additionally, positive IgG Ab for *Leptospira*, dengue, and *S. typhi* was also recorded. The test for Leishmanial (K‐39) Ab was negative, but the test for Total *Brucella* Ab agglutination was positive, indicating exposure to the bacteria but not establishing acute or previous infection. One hundred milligram of doxycycline was immediately started and given daily two times a day. Serological testing and subsequent hematological as well as the biochemical profile is documented in Tables 1 and 2.

The patient received regular follow‐ups and no changes were made to the prescription. Follow‐up showed no improvement in the liver enzyme levels – alkaline phosphatase value of >1600 U/L; bilirubin levels had dropped from the first follow‐up but had remained clinically higher. Low hemoglobin level, low hematocrit %, low red blood cell indices, lymphocytosis, neutropenia, increase in bilirubin, and liver enzymes were seen throughout the follow‐up course. Platelets level fluctuated during the course but eventually returned to normal. The patient was eventually discharged on Day 10 when she was hemodynamically stable and symptomatically better. All the follow‐up laboratory investigations performed until discharge was represented in Table 2.

The patient received a comprehensive description of the proper usage of the prescribed medication. Tab rifampicin 600 mg was prescribed once a day daily for 5 weeks and 1 day. Likewise, Tab doxycycline 100 mg was prescribed for 5 weeks, two times a day. Additionally, 500 mg of paracetamol was prescribed to be taken as needed. The patient was informed upon discharge of any potential warning indications. It was advised to stay hydrated and to use insect repellant and/or mosquito nets.

## 3 DISCUSSION

The case illustrates the acute bacterial co‐infection of *S. typhi* and *O. tsutsugamushi* in a patient with dengue fever. Since we used a rapid serology test kit for diagnosis, there were some questions raised about their accuracy. Due to the lack of resources in the settings and the deteriorating condition of the patient, the need for a quick diagnostic approach was needed. Clinical laboratory parameters also played a vital role in taking medical decisions in addition to rapid serological tests. Other tests having more diagnostic sensitivity and specificity such as ELISA and/or polymerase chain reaction would lead to more confirmatory identification but were not accessible in our setup. Since confirmatory tests were not readily accessible and rapid turnaround time was crucial for proper patient management, there is a possibility of false positivity.

Dengue infection was diagnosed based on IgM positivity. The manufacturer (SD Bioline™ Dengue Duo; Abbott) reported a sensitivity of 94.2% and specificity of 96.4% for the diagnosis of acute IgM dengue infection. *S. typhi* IgG/IgM (Bioline Diagnostics LLP) was also identified based on IgM positivity with sensitivity and specificity of 91% and 99.3%, respectively. Suspecting the possible cross‐reactivity of Rapid *S. typhi* IgM in dengue fever, which is also concluded with the study performed by Bhatti et al.,^5^ a follow‐up test was requested. Three to four days following the initial test, a follow‐up test showed that both the IgG/IgM tests for *S. typhi* and dengue were IgG‐positive. The study performed by Bhatti et al.^5^ concluded cross‐reactivity based on positive *S. typhi* IgM but negative IgG on dengue patients using rapid kits, but our test showed positive IgM‐Ab on the initial test and subsequent IgG positivity on follow‐up. ImmuneMed Scrub Typhus Rapid test kit was used for the qualitative detection of IgM/IgG Ab specific to *O. tsutsugamushi*. The sensitivity and specificity were 97.3% and 99.7%, respectively.

Various studies and case reports have been documented regarding the co‐infection of dengue virus and scrub typhus,^6^ scrub typhus and enteric fever^7^ as well as dengue and enteric fever^8^ in Nepal. Few cases of all three, that is, dengue, scrub typhus, and *S. typhi* co‐infection was reported in India.^9^

Our patient worked primarily in agricultural fields in the Dang district of Nepal. Her work may have had her exposed to ticks and mites. After the 2015 earthquake, scrub typhus emerged in Nepal, and the disease is diagnosed usually following heavy rainfall starting in July until the post‐monsoon season of November.^10^ In this case study, the patient presented first symptoms onset during September which marked the end of monsoon season. Similar to our patient study, Murdoch et al.^7^ also did not observe any eschar in a patient with scrub typhus‐typhoid co‐infection. Furthermore, the clinical symptoms of the patient were relatively stable when doxycycline was administered. Therefore, on discharge, rifampicin and doxycycline were prescribed to the patient which are the most potent drugs to treat mild scrub‐typhus infection.^11^ Additionally, thrombocytopenia, transaminitis, and hyperbilirubinemia along with subsequent hypoalbuminemia were seen in our patient which is also consistent with dengue‐scrub infection as conducted by Basheer et al.^12^ Another study in the scrub‐typhoid and dengue‐typhoid co‐infection revealed low hemoglobin levels, thrombocytopenia, and an increase in transaminases level which is also consistent with our findings.^12^, ^13^

Our patient presented co‐infection with *S. typhi* and scrub typhus in midst of the dengue outbreak making proper treatment challenging. As acetaminophen such as paracetamol is the widely recommended drug for dengue infection, the patient was administered with proper dosage of paracetamol orally. However, as the patient was also found to be positive for Salmonella IgM, ceftriaxone was administered immediately. A case report of dengue‐typhoid co‐infection in a child by Srinivasaraghavan et al.^13^ also treated a patient with ceftriaxone and other drugs to which the patient responded fairly and then discharged 10 days later. On follow‐up tests, scrub‐typhus IgM was found to be positive, so the patient was started on doxycycline, an effective drug used in a case of dengue‐typhus co‐infection.^6^

There is a high chance to get treatment failure in co‐infection cases; therefore, it requires accurate identification of the etiology. On the other hand, correct treatment and proper patient management is also a critical challenge for physicians. Thus, in the case of resource‐limited settings, patient management via rapid turnaround time of diagnostic tests plays an important role.

The preprint of this article can be found on authorea https://www.authorea.com/users/520091/articles/593539‐co‐infection‐of‐dengue‐scrub‐typhus‐and‐typhoid‐during‐dengue‐outbreak‐in‐nepal‐2022‐a‐case‐report.

## 4 CONCLUSION

Nepal is prevalent to numerous endemic diseases such as dengue, scrub typhus, and typhoid fever. Thus, multiple co‐infections may result in a febrile sickness with an overlapping spectrum of symptoms that can be difficult to detect and treat. Rapid diagnostic tests, as well as subsequent correlation with other laboratory parameters, can be useful in the early treatment of a patient and subsequently decrease the burden of co‐infection and mortality.

## AUTHOR CONTRIBUTIONS

**Bibek Raj Bhattarai:** Data curation; formal analysis; investigation; writing – original draft; writing – review and editing. **Rajshree Bhujel:** Supervision; writing – original draft; writing – review and editing. **Sushant Pokhrel:** Writing – original draft; writing – review and editing. **Abhishek Mishra:** Formal analysis; investigation; writing – original draft. **Anamika Priyadarshinee:** Data curation; investigation; supervision; validation; visualization.

## FUNDING INFORMATION

The author(s) received no financial support for the research, authorship, and/or publication of this article.

## CONFLICT OF INTEREST

The author(s) declared no potential conflicts of interest.

## ETHICAL APPROVAL

Our institution does not require ethical approval for reporting individual cases.

## CONSENT

Written informed consent was obtained from the patient to publish this report in accordance with the journal's patient consent policy.

## Data Availability

Data sharing not applicable to this article as no datasets were generated or analysed during the current study.
